# Response-Adapted Treatment Following Radiotherapy in Patients With Resectable Locally Advanced Hypopharyngeal Carcinoma

**DOI:** 10.1001/jamanetworkopen.2022.0165

**Published:** 2022-02-22

**Authors:** Xi Luo, Xiaodong Huang, Shaoyan Liu, Xiaolei Wang, Jingwei Luo, Jianping Xiao, Kai Wang, Yuan Qu, Xuesong Chen, Ye Zhang, Jingbo Wang, Jianghu Zhang, Guozhen Xu, Li Gao, Runye Wu, Junlin Yi

**Affiliations:** 1 Department of Radiation Oncology, National Cancer Center/National Clinical Research Center for Cancer/Cancer Hospital, Chinese Academy of Medical Sciences and Peking Union Medical College, Beijing, China; 2 Department of Head and Neck Surgical Oncology, National Cancer Center/National Clinical Research Center for Cancer/Cancer Hospital, Chinese Academy of Medical Sciences and Peking Union Medical College, Beijing, China

## Abstract

**Importance:**

Laryngeal preservation strategies for resectable locally advanced hypopharyngeal carcinoma (LAHPC) have been explored. However, the optimal strategy remains unclear.

**Objective:**

To evaluate a response-adapted strategy based on an early response to radiotherapy (RT) in patients with resectable LAHPC.

**Design, Setting, and Participants:**

This cohort study was conducted from May 2009 to October 2019 with a median (IQR) follow-up period of 66.5 (44.7-97.0) months. The study was conducted at a tertiary academic medical center and included 423 patients pathologically confirmed stage III and IVB LAHPC. A total of 250 patients with previous cancer history, synchronous primary cancer, stage I or II, or with unresectable hypopharyngeal carcinoma were excluded.

**Exposures:**

Patients who reached 80% or greater tumor regression when evaluated endoscopically and by imaging methods at 50 Gy received definitive RT or concurrent chemoradiotherapy, and those with less than 80% regression underwent surgery 4 to 6 weeks after RT.

**Main Outcomes and Measures:**

Five-year overall survival and survival with a functional larynx.

**Results:**

Overall, 423 patients were included in the study (median [IQR] age, 55 [50-63] years; 408 [96.5%] men and 15 [3.5%] women). The response-adapted and primary surgery groups had significantly better survival than the primary RT group (52.7% and 54.4% vs 27.7%, respectively; *P* < .001). The response-adapted and primary surgery groups had similar 5-year overall survival of 52.7% vs 54.4%, respectively (hazard ratio [HR], 1.02; 95% CI, 0.75 to 1.39; *P* = .89). The response-adapted group had better 5-year survival with functional larynx than the primary surgery group (40.6% vs 33.9%; HR, 0.64; 95% CI, 0.49 to 0.84, *P* = .001). Surgery complications did not significantly differ between the 2 groups. Among patients in the response-adapted group who required total laryngectomy (n = 186) as indicated by pretreatment evaluation, the 5-year cumulative Kaplan-Meier survival with functional larynx was 39.8%.

**Conclusions and Relevance:**

In this cohort study, the response-adapted strategy based on an early RT response facilitated better treatment tailoring, maximum tumor control, and higher laryngeal preservation compared with primary surgery and primary RT strategies. This approach could provide a feasible laryngeal preservation strategy in patients with LAHPC.

## Key Points

**Question:**

Is a response-adapted strategy based on early tumor response to radiotherapy associated with improved survival with a functional larynx in patients with resectable locally advanced hypopharyngeal carcinoma?

**Findings:**

In this cohort study of 423 patients with resectable locally advanced hypopharyngeal carcinoma, a response-adapted strategy based on an early tumor response to radiotherapy improved survival with a functional larynx compared with primary surgery and primary radiotherapy strategies.

**Meaning:**

These findings suggest that a response-adapted strategy based on an early tumor response to radiotherapy could be considered a feasible laryngeal preservation strategy.

## Teaser

This cohort study compares survival outcomes following response-adapted treatment strategy with outcomes following primary surgery and primary radiotherapy strategies among patients with locally advanced hypopharyngeal carcinoma.t

## Introduction

Hypopharyngeal carcinoma (HPC) has one of the poorest prognoses of head and neck squamous cell carcinomas.^1,2^ Considering its prognosis and the adjacent functional structures in affected patients, survival and organ preservation are both important in patients with HPC.^3,4^ For early-stage HPC, surgery or radiotherapy (RT) can both result in a favorable prognosis. However, the treatment of locally advanced hypopharyngeal carcinoma (LAHPC) remains challenging, with a reported 5-year overall survival (OS) rate of 30% to 40% and most patients requiring total laryngectomy.^5,6,7^ Since the 1980s, many studies^7,8,9,10,11,12,13,14,15,16,17,18^ have attempted to explore laryngeal-preservation strategies in patients with locally advanced laryngeal cancer and LAHPC. As a result, 2 laryngeal-preservation approaches have been established: (1) induction chemotherapy (IC) followed by RT or concurrent chemoradiotherapy (CCRT) and (2) CCRT.

In the case of IC followed by RT or CCRT, IC is used to select good candidates to receive radical RT or CCRT, and others undergo surgery.^7,10^ This strategy is based on the correlation between radiosensitivity and chemosensitivity.^19,20^ However, chemosensitivity cannot directly represent radiosensitivity. The European Organization for Research and Treatment of Cancer,^10^ which studied this approach, found a 5-year survival with a functional larynx (SFL) of 22%. Beijing Tongren Hospital conducted a large prospective observational cohort study^21^ involving the administration of 2 cycles of therapy with paclitaxel, cisplatin, and 5-fluorouracil to select patients who respond well to RT to receive radical CCRT and those who do not to undergo surgery. In the study, 5-year OS and laryngoesophageal dysfunction–free survival of 32.6% and 24.8%, respectively, were achieved. However, in these studies that used IC to select patients for subsequent treatment, OS and SFL remained unfavorable.

CCRT has become a standard approach for the management of locally advanced head and neck squamous cell cancer.^6,8,9^ Nevertheless, this strategy means that patients receive radical CCRT without a selection process, which may adversely affect OS and SFL in patients with LAHPC. Moreover, some studies even indicated that surgical resection remains necessary to achieve maximum tumor control and functional preservation.^22,23^ Compared with laryngeal carcinoma, LAHPC is associated with worse survival and a higher risk of salvage surgery. In the case of LAHPC, salvage surgery usually means a success rate of 40% to 50% and a wound complication risk of 50% to 80%.^2,24,25,26,27^ Thus, the optimal timing of surgery, which can maximize tumor control without increasing the risk of wound complications for LAHPC, is important.

In some studies, patients who responded poorly to IC achieved a good prognosis after undergoing radical RT or CCRT.^7,11^ Favorable early responses to RT usually lead to better local control and survival in patients with head and neck squamous cell carcinomas and other cancers.^28,29,30^ Several attempts have been made to select patients based on early tumor responses to RT, which can directly represent the radiosensitivity of LAHPC. The response-adapted strategy includes patients showing more than 80% tumor regression who received RT or CCRT and those showing less than 80% regression who received surgery 4 to 6 weeks after RT or CCRT. The response-adapted strategy has been associated with better survival, laryngeal preservation, and an acceptable toxicity profile^31^ compared with studies in which IC was used to select patients.^7,10^

Thus, it is necessary to assess the response-adapted strategy based on early tumor responses to RT in a large cohort. To this end, we assessed clinical outcomes for patients with LAHPC and sought to explore a more effective laryngeal-preservation strategy for resectable LAHPC using a large cohort of patients with LAHPC.

## Methods

This study followed the Strengthening the Reporting of Observational Studies in Epidemiology (STROBE) reporting guideline was followed in this study. The study was approved by the institutional review board at the National Cancer Center/Cancer Hospital, Chinese Academy of Medical Sciences and Peking Union Medical College, which waived the need for informed consent because patient data were deidentified in the data set.

### Patient Population

**Figure 1.**
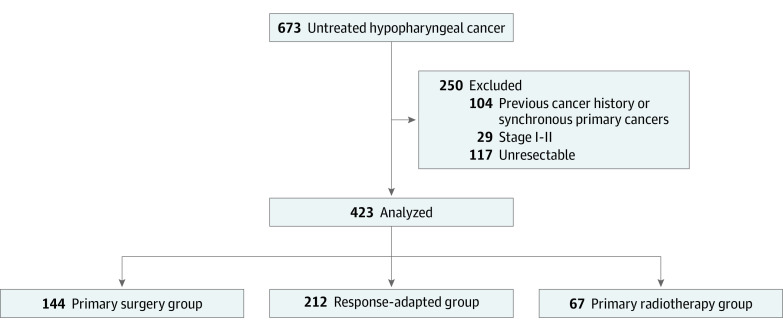
Enrollment Diagram

In this analysis, data for 423 newly diagnosed patients with resectable, stage III and IVB HPC between May 2009 and October 2019 were reviewed (Figure 1). All patients underwent comprehensive staging procedures according to the American Joint Committee on Cancer 8th Edition (AJCC).^32^ Extranodal extension (ENE) was evaluated based on unambiguous evidence of gross ENE.

### Response-Adapted Strategy

The response-adapted strategy was determined based on the primary tumor response, which was evaluated at a dose of 50 Gy. All staging procedures were repeated at a dose of 50 Gy. If the response reached complete response or partial response (more than 80% tumor regression), patients received radical RT or CCRT; otherwise, they received surgery, if possible, at 4 to 6 weeks after RT. All patients who reached partial response at 50 Gy were defined as responsive; the rest were defined as nonresponsive (eFigure 1 in the Supplement).

### Treatment

All primary treatment regimens were determined on the advice of the head and neck multidisciplinary team advice and the preference of the patient. The head and neck multidisciplinary team assessed the resectability of the tumors and the need for total laryngectomy before treatment (total laryngectomy was needed once the tumors invaded the interarytenoid notch or the postcricoid area close to the central line or showed extension to the esophagus).

The primary surgery group (n = 144) underwent surgery primarily with or without postoperative RT or CCRT. Patients in the primary RT group (n = 67) received radical RT or CCRT. Patients in the response-adapted group underwent the response-adapted strategy (n = 212). These patients received standard-fractionated RT (1.82-2.12 Gy per day, 5 days per week) with 70 Gy to the gross tumor volume, 66 Gy to the tumor-bed area, 60 Gy to the high-risk clinical target volume, and 50 Gy to prophylactic region.

Patients aged 18 to 70 years who showed good Eastern Cooperative Oncology Group (ECOG) scores; adequate hematological, hepatic, and kidney function and no severe comorbidities usually received RT concomitantly with platinum-based chemotherapy. Patients with positive surgical margins and ENE status also usually received postoperative CCRT. The most common chemotherapy regimen involved concomitant cisplatin administration at a dose of 100 mg/m^2^ every 3 weeks. Additionally, IC was not conventionally used in our center, and only a few patients with high tumor burden received IC. Nine patients (6.3%) in the primary surgery group received IC. In contrast, the response-adapted group and primary RT group had similar proportions of patients receiving IC (24.5% and 22.4%, respectively). The most commonly used regimen consisted of paclitaxel, cisplatin, and 5-fluorouracil.

### Statistical Analysis

The end points were OS (defined as the duration from treatment to death from any cause), progression-free survival (PFS; until progression, relapse, or death) and SFL (based on the strictest definition, in which failure was defined as death from any cause and total laryngectomy, local relapse or progression, or the need for a tracheotomy or feeding tube, whichever occurred first).

We assessed tumor response in accordance with response evaluation criteria in Solid Tumors version 1.1.^33^ Toxicity was classified and scaled according to the European Organization for Research and Treatment of Cancer radiation morbidity scoring criteria.^34^ Survival data were estimated using the Kaplan-Meier method. A Cox regression model was used to identify risk factors for survival. Propensity-score matching (PSM) was conducted to balance prognostic factors and generate comparable study arms. Two-sided *P* < .05 was considered significant.

## Results

### Clinical Features

A total of 423 patients were included in the study (median [IQR] age, 55 [50-63] years; 408 [96.5%] men and 15 [3.5%] women). The proportion of ENE was lower in the primary surgery group (13 patients [9.0%]). Thus, more cases were classified into the IVB stage in the response-adapted group (47 patients [22.2%]) and primary RT group (20 patients [29.9%]) according to AJCC. The clinical characteristics of this cohort are shown in Table 1.

**Table 1.** Baseline Characteristics of the Groups

Characteristic	No. (%)				*P* value
	Total (N = 423)	Primary surgery group (N = 144)	Response-adapted group (N = 212)	Primary RT group (N = 67)	
Sex					.49
Female	15 (3.5)	4 (2.8)	7 (3.3)	4 (6.0)	
Male	408 (96.5)	140 (97.2)	205 (96.7)	63 (94.0)	
Age, y					.11
>56	209 (49.4)	78 (54.2)	94 (44.3)	37 (55.2)	
≤56	214 (50.6)	66 (45.8)	118 (55.7)	30 (44.8)	
ECOG					.24
0	51 (12.0)	22 (15.2)	24 (11.3)	5 (7.5)	
1	370 (87.5)	121 (84.1)	188 (88.7)	61 (91.0)	
≥2	2 (0.5)	1 (0.7)	0	1 (1.5)	
Pathological type					.52
SC	417 (98.6)	142 (98.6)	208 (98.1)	67 (100.0)	
Other	6 (1.4)	2 (1.4)	4 (1.9)	0	
Subsite					<.001
PS	337 (79.7)	98 (68.1)	181 (85.4)	58 (86.5)	
PPW	64 (15.1)	40 (27.8)	20 (9.4)	4 (6.0)	
PC	22 (5.2)	6 (4.2)	11 (5.2)	5 (7.5)	
ENE	77 (18.2)	13 (9.0)	46 (21.7)	18 (26.9)	.001
cT stage (AJCC 7th/8th)					.04
T1-2	129 (30.5)	52 (36.1)	64 (30.2)	13 (19.4)	
T3	121 (28.6)	39 (27.1)	66 (31.1)	16 (23.9)	
T4a	173 (40.9)	53 (36.8)	82 (38.7)	38 (56.7)	
cN stage (AJCC 7th)					.15
N0	56 (13.2)	26 (18.1)	25 (11.8)	5 (7.5)	
N1	42 (9.9)	15 (10.4)	20 (9.4)	7 (10.4)	
N2	302 (71.4)	100 (69.4)	152 (71.7)	50 (74.6)	
N3	23 (5.5)	3 (2.1)	15 (7.1)	5 (7.5)	
cN stage (AJCC 8th)					.01
N0	56 (13.3)	26 (18.1)	25 (11.8)	5 (7.5)	
N1	42 (9.9)	15 (10.4)	20 (9.4)	7 (10.4)	
N2	245 (57.9)	90 (62.5)	120 (56.6)	35 (52.2)	
N3	80 (18.9)	13 (9.0)	47 (22.2)	20 (29.9)	
cStage (AJCC 7th)					.04
III	58 (13.7)	26 (18.0)	28 (13.2)	4 (6.0)	
IVA	342 (80.9)	115 (79.9)	169 (79.7)	58 (86.5)	
IVB	23 (5.4)	3 (2.1)	15 (7.1)	5 (7.5)	
cStage (AJCC 8th)					.002
III	58 (13.7)	26 (18.1)	28 (13.2)	4 (6.0)	
IVA	284 (67.1)	104 (72.2)	137 (64.6)	43 (64.1)	
IVB	81 (19.2)	14 (9.7)	47 (22.2)	20 (29.9)	
Pretreatment evaluation					<.001
Total laryngectomy	313 (74.0)	65 (45.1)	186 (87.7)	62 (92.5)	
Laryngeal-preservation surgery	110 (26.0)	79 (54.9)	26 (12.3)	5 (7.5)	
Concurrent chemotherapy^a^	222 (59.5)	38 (40.4)	143 (67.5)	41 (61.2)	<.001
Radiation technology^a^					.10
IMRT	356 (95.4)	88 (93.6)	207 (97.6)	62 (92.5)	
2-D RT/3-D CRT	34 (9.1)	6 (6.4)	5 (2.4)	5 (7.5)	
Received salvage surgery	34 (8.0)	2 (1.4)	22 (10.4)	10 (14.9)	.001

Abbreviations: 2-D, 2-dimensional; 3-D, 3-dimensional; AJCC, American Joint Committee on Cancer; CRT, conformal radiotherapy; cN, clinical N stage; cT, clinical T stage; ECOG, Eastern Cooperative Oncology Group; ENE, extranodal extension; IMRT, intensity-modulated radiotherapy; PC, postcricoid; PPW, posterior pharyngeal wall; PS, pyriform sinus; RT, radiotherapy; SC, squamous cell carcinoma.

^a^ Among patients who received RT (n = 373).

### Survival

The median (IQR) follow-up period was 66.5 (44.7-97.0) months. The 5-year OS, PFS, and SFL of the whole cohort were 49.5%, 43.5%, and 36.3%, respectively. The 5-year OS, PFS, and SFL according to the AJCC system were, respectively, as follows: stage III (n = 58), 58.8%, 55.3%, and 44.4%; stage IVA (n = 284), 54.6%, 47.0%, and 38.7%; and stage IVB (n = 81), 25.8%, 22.9%, and 22.0%.

**Figure 2.**
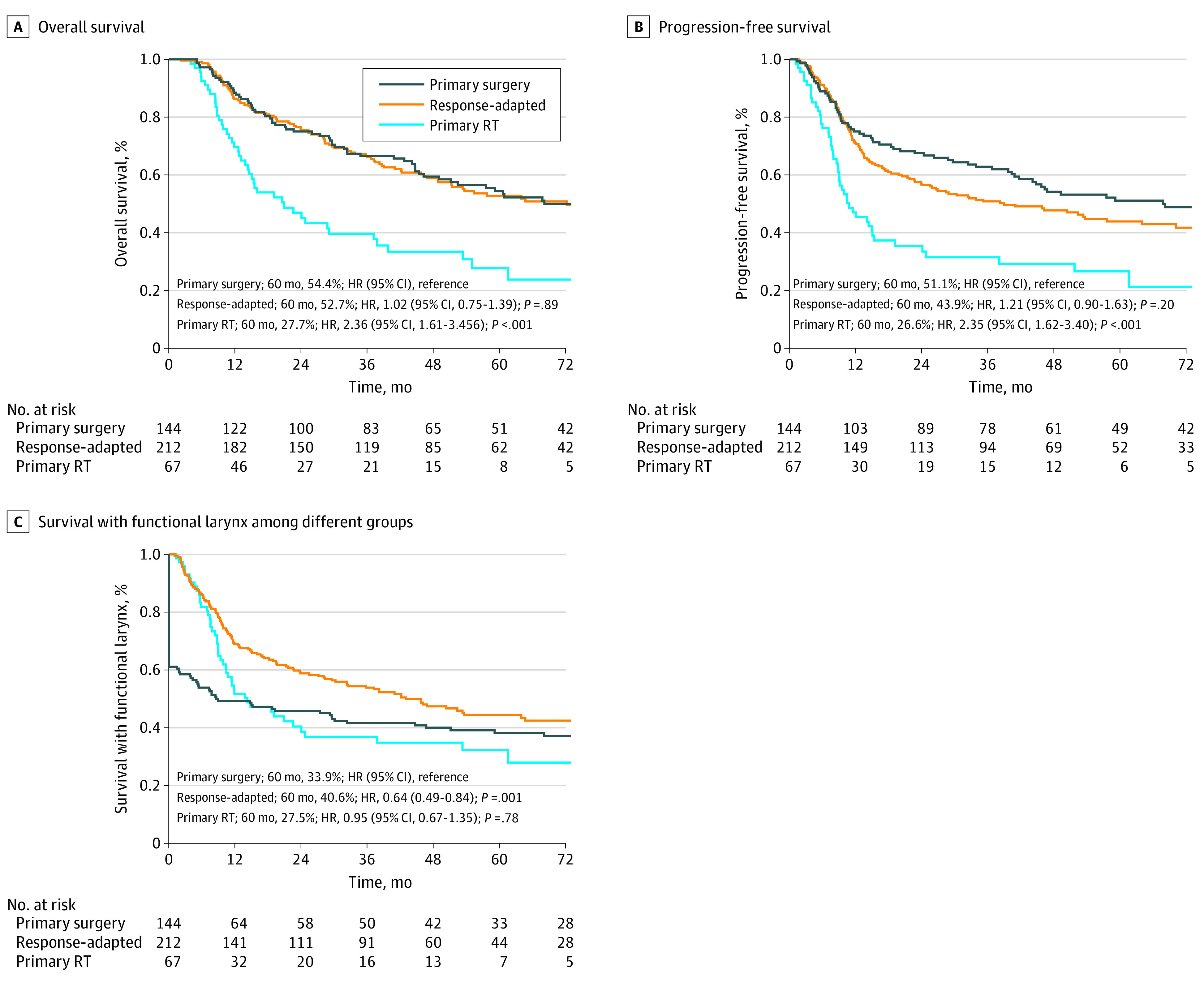
Kaplan-Meier Survival Curves by Treatment Strategy HR indicates hazard ratio; RT, radio therapy.

In evaluations based on the different treatment strategies, the 5-year OS, PFS, and SFL were, respectively, 54.4%, 51.1%, and 33.9% in the primary surgery group (n = 144); 52.7%, 43.9%, and 40.6% in the response-adapted group (n = 212); and 27.7%, 26.6%, and 27.5% in the primary RT group (n = 67). No significant differences were observed between the primary surgery and response-adapted groups at OS and PFS. However, these 2 strategies were associated with better survival than primary RT. OS, PFS, and SFL among the 3 groups are shown in Figure 2. In the primary RT group, 41 of 67 patients (61.2%) received radical CCRT, and the 5-year OS, PFS, and SFL of these patients were 36.0%, 33.0%, and 36.3%, respectively.

Of the 423 patients, 76 received induction chemotherapy (IC) and 18 were nonresponsive after 2 cycles of IC. Among the 18 patients who were nonresponsive to IC, 12 (66.6%) received radical RT, and 6 of these 12 patients achieved long-term survival.

### Laryngeal Preservation

**Figure 3.**
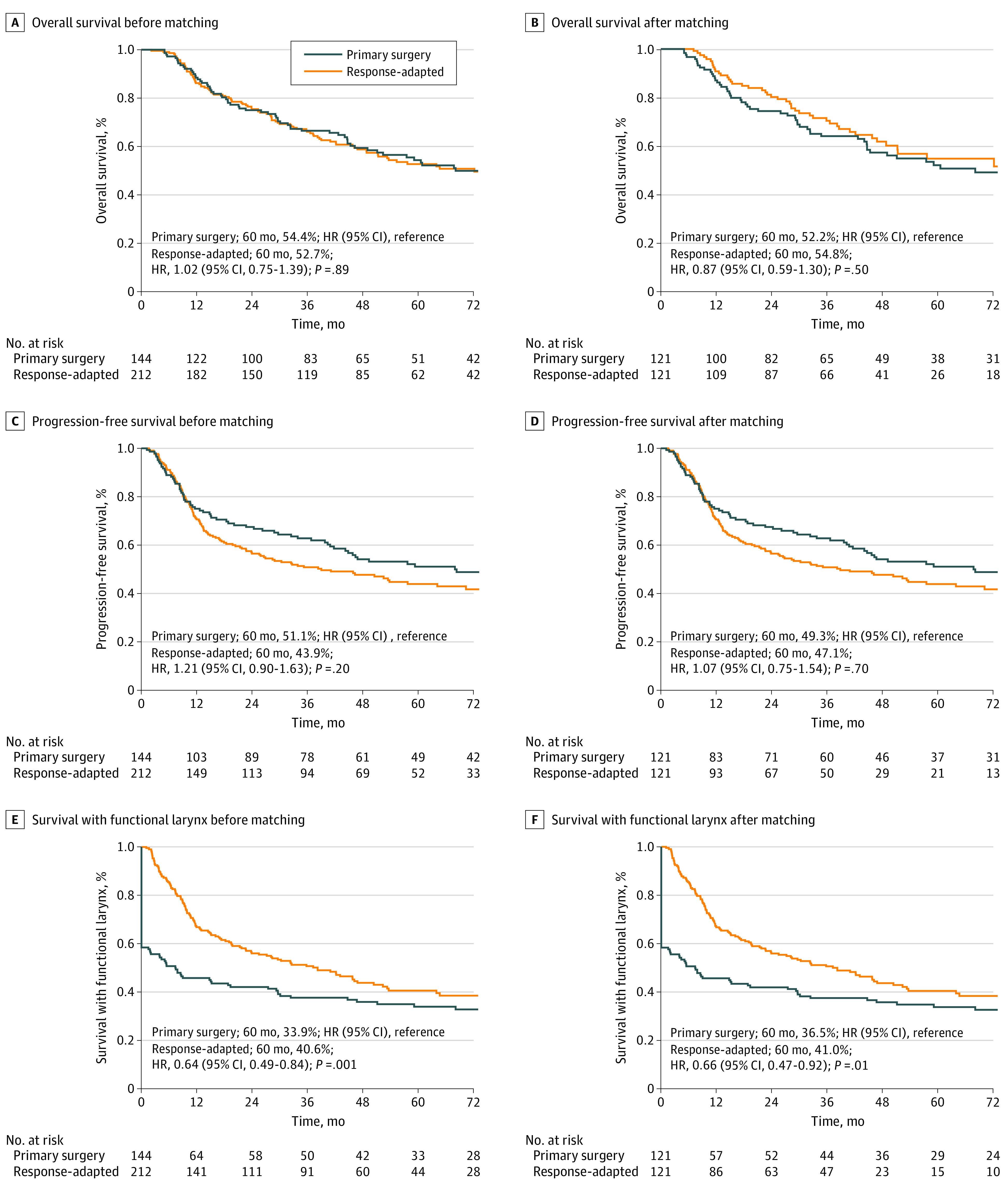
Kaplan-Meier Survival Curves Before and After Matching HR indicates hazard ratio.

The median (IQR) follow-up duration was 66.5 (43.9-80.1) months for the primary surgery group and 77.8 (47.5-111.8) months in the response-adapted strategy group. The response-adapted strategy group showed a similar unadjusted 5-year OS as the primary surgery group, with rates of 52.7% vs 54.4% (hazard ratio [HR], 1.02; 95% CI, 0.75-1.39; *P* = .89; Figure 3A). The unadjusted 5-year PFS rates in these 2 groups were 43.9% and 51.1% (HR, 1.21; 95% CI, 0.90-1.63, *P* = .20; Figure 3C), respectively. The 5-year SFL of the response-adapted group was 40.6% and that of the primary surgery group was 33.9% (HR, 0.64; 95% CI, 0.49-0.84; *P* = .001; Figure 3E).

A subgroup analysis was performed among patients without induction chemotherapy (n = 347) (eFigure 2 in the Supplement). The response-adapted group still showed a better 5-year OS and SFL than the primary surgery and primary RT groups.

Because the baseline data between the primary surgery group and response-adapted group were unbalanced, we used 6 factors (sex, ECOG, ENE, T stage, N stage, and TNM stage) to balance the 2 groups (121 patients in each group; eTable 1 in the Supplement). Adjusted 5-year OS and PFS were 54.8% and 47.1%, respectively, in the response-adapted group vs 52.2% (HR, 0.87; 95% CI, 0.59-1.30, *P* = .50; Figure 3B) and 49.3% (HR, 1.07; 95% CI, 0.75-1.54, *P* = .70; Figure 3D), respectively, in the primary surgery group. Besides, the adjusted 5-year SFL in the response-adapted group was 41.0% compared with 36.5% (HR, 0.66; 95% CI, 0.47-0.92, *P* = .01; Figure 3F) in the primary surgery group.

The proportion of patients who needed total laryngectomy in the response-adapted group in the pretreatment evaluation was 87.7% (186 of 212), while the corresponding value was 45.1% (65 of 144) in the primary surgery group (*P* < .001). Among the 186 patients who required total laryngectomy in the pretreatment evaluation before the response-adapted strategy, the 5-year SFL was 39.8%.

### Treatment Complications

A total of 144 patients received surgery in the primary surgery group and 46 patients received surgery after 50 Gy in the response-adapted group. The rates of surgical complications were 22.9% (33 of 144) in the primary surgery group and 32.6% (15 of 46) (*P* = .19) in the response-adapted group. Pharyngeal fistula was the most common complication, and there was no significant difference in the incidence of this complication between these 2 groups, with rates of 20.1% (29 of 144) vs 28.3% (13 of 46) (*P* = .25), respectively. The mean (SD) duration of hospitalization was 19.1 (6.1) days in the primary surgery group and 18.0 (7.1) days in the response-adapted group.

### Treatment Failure

By the end of the last follow-up visit, 190 of 423 patients (44.9%) developed treatment failure, most of which (154 [81.0%]) occurred within 2 years. In the entire failure cohort, 95 patients (22.5%) experienced local recurrence, 61 (14.4%) developed regional recurrence, and 87 (20.6%) showed distant metastasis. Local and regional recurrence was the main failure pattern in resectable locally advanced HPC.

### Prognostic Factors for Survival

In multivariable analysis, T stage and N stage were independently significant for OS, PFS, and SFL (Table 2). In addition, treatment strategies remained an independent prognostic factor for OS, PFS, and SFL, favoring a response-adapted strategy.

**Table 2.** Multivariate Analysis Results of Clinical Variables and Treatment Affecting Survival and Larynx Preservation

Variable	OS		PFS		SFL	
	HR (95% CI)	*P* value	HR (95% CI)	*P* value	HR (95% CI)	*P* value
ECOG						
1 vs 0	2.20 (1.25-3.88)	.007	2.00 (1.21-3.29)	.007	1.30 (0.86-1.99)	.23
Subsite						
PC vs PS	1.27 (0.68-2.37)	.45	1.12 (0.62-2.02)	.72	1.28 (0.75-2.19)	.36
PPW vs PS	1.45 (1.00-2.10)	.05	1.45 (1.01-2.09)	.04	1.47 (1.05-2.05)	.03
cT						
T3 vs T1-2	1.83 (1.22-2.76)	.004	1.88 (1.30-2.73)	<.001	2.11 (1.46-3.06)	<.001
T4a vs T1-2	2.11 (1.48-3.00)	<.001	1.95 (1.41-2.70)	<.001	3.64 (2.60-5.10)	<.001
cN						
N1 vs N0	0.97 (0.50-1.89)	.93	0.95 (0.51-1.77)	.87	0.60 (0.34-1.04)	.07
N2 vs N0	1.24 (0.80-1.93)	.33	1.38 (0.91-2.08)	.13	0.88 (0.61-1.26)	.47
N3 vs N0	2.48 (1.51-4.08)	<.001	2.57 (1.61-4.11)	<.001	1.24 (0.81-1.88)	.32
Treatment strategy						
Response-adapted vs primary surgery	0.95 (0.69-1.31)	.76	1.14 (0.84-1.55)	.40	0.50 (0.38-0.67)	<.001
Primary RT vs primary surgery	1.82 (1.22-2.73)	.004	2.02 (1.37-2.97)	<.001	0.65 (0.45-0.95)	.03

Abbreviations: cN, clinical N stage; cT, clinical T stage; ECOG, Eastern Cooperative Oncology Group; HR, hazard ratio; OS, overall survival; PC, postcricoid; RT, radiotherapy; PFS, progression-free survival; PPW, posterior pharyngeal wall; PS, Pyriform sinus; SFL, survival with functional larynx.

## Discussion

In this study, the response-adapted strategy based on early tumor response to RT achieved excellent survival and laryngeal-preservation outcomes for resectable LAHPC in comparison with historical studies that used IC to select patients.^7,10^ Although the patients in the primary surgery group had an earlier tumor stage and included a higher proportion of patients who could receive laryngeal-preservation surgery in the pretreatment evaluation, the response-adapted group still showed significantly better laryngeal preservation and had equal survival without the additional treatment complications in comparison with the primary surgery group. The primary RT group showed the worst survival and SFL among the 3 groups. Moreover, among the 186 patients who were evaluated as requiring total laryngectomy before treatment in the response-adapted group, a favorable 5-year SFL of 39.8% was achieved.

Several laryngeal-preservation strategies have been investigated since the 1990s^7,8,9,10,11,12,13,14,15,16,17,18^ (eTable 2 in the Supplement). The European Organization for Research and Treatment of Cancer trial^7,10^ validated induction PF followed by RT as a feasible strategy for laryngeal preservation without reducing survival Additionally, several studies tried to explore a new IC regimen and add cetuximab in the laryngeal-preservation strategy and showed improvements.^12,13,14,15,16,35^ Beijing Tongren Hospital used 2 cycles of paclitaxel, cisplatin, and 5-fluorouracil in a large prospective observational cohort to select patients to receive radical CCRT.^21^ That study achieved 5-year OS and laryngoesophageal dysfunction–free survival of 32.6% and 24.8%, respectively.^21^ However, some laryngeal preservation studies^12,13,14,15,16^ included 40% to 50% patients with laryngeal carcinoma, which had much better outcomes than LAHPC.^36^ In our study, we achieved significantly better 5-year OS and SFL of 54.8% and 41.0% (after adjustment), respectively, in the response-adapted group than those reported with the use of IC to select patients.^7,8,9,10,11,12,13,14,15,16,17,18^

Although radical CCRT has been widely used in locally advanced head and neck squamous cell cancer, some studies^22,23^ indicate that salvage surgical resection for residual and recurrent lesions is still necessary to maximize tumor control and functional preservation in locally advanced head and neck squamous cell cancer. The main failure pattern of LAHPC in this study was local-regional failure, suggesting that improvement in local-regional control may contribute to prognosis. With the primary RT strategy, all patients received radical RT without selection, and while patients who were responsive to RT could achieve good prognoses, those who were not responsive to RT usually showed lower local-regional tumor control and unfavorable survival outcomes. In contrast, the response-adapted strategy used the early response to RT or CCRT to identify patients who were responsive and subsequently received radical RT or CCRT and those who were not responsive, who received surgery. With this approach, the response-adapted group showed a significantly better OS of 52.7% in comparison with 27.7% (*P* < .001) in the primary RT group. The response-adapted group showed a similar OS and PFS and a significantly higher survival with a functional larynx in comparison with the primary surgery group (after adjustment). These findings suggest that the optimal timing of surgery may play an important role in achieving maximum tumor control and functional preservation.

For a long time, primary surgery was a prominent option in locally advanced HPC, with a reported 5-year OS of 30% to 50%.^7,10,37,38^ After adjustment, the 5-year OS and PFS were similar in the primary surgery group and the response-adapted group. Although the proportion of patients who needed total laryngectomy when evaluated before treatment in the response-adapted group (87.7%) was higher than that in the primary surgery group (45.1%), the 5-year SFL in the response-adapted group was 41.0%, which was better than the corresponding value in the primary surgery group (36.5%; *P* = .01, after adjustment). Moreover, in the subset of 186 patients in the response-adapted group who were evaluated as needing total laryngectomy, the 5-year SFL was still 39.8%. These survival and laryngeal preservation rates demonstrated benefit with the response-adapted strategy in resectable LAHPC in this study.

The main failure pattern in this study was local and regional recurrence, and surgical resection was still necessary to achieve maximum tumor control and superior survival in locally advanced head and neck squamous cell cancer.^22,23^ Salvage surgery usually involves high positive resection margin rates of 12% to 40% and a high risk of pharyngeal fistula (11% to 58%).^2,24,25,26,27^ However, in our study, although the rates of surgical complications and pharyngeal fistulas in the response-adapted and primary surgery groups were 32.6% vs 22.9% and 28.3% vs 20.1%, respectively, the differences were not significant. Thus, the laryngeal preservation and similar rates of surgical complications in the response-adapted strategy group demonstrate that the timing of surgical intervention based on the tumor response at DT 50 Gy was favorable and feasible.

### Limitations

This study had limitations. Although the data confirmed favorable outcomes with the response-adapted strategy, the treatments in different groups were not randomly assigned. Patients who refused surgery or were unfit for surgery were treated with radical RT or CCRT; this favored the surgical group because patients unfit for surgery were likely to have poorer treatment outcomes. These selection biases may have affected our results. We attempted to use PSM to circumvent this limitation, and the numbers of patients (ie, more than 100) were sufficient to compare the primary surgery and response-adapted groups. However, since the primary RT group contained only 67 patients, we did not use PSM to compare the primary RT group with the other 2 groups.

## Conclusions

In summary, the response-adapted strategy based on early tumor response to RT was associated with better tumor control and laryngeal preservation in comparison with the other strategies. In comparison with the primary surgery group, the response-adapted strategy group achieved equal oncological outcomes, superior laryngeal preservation, and no additional operative complications. Thus, the response-adapted strategy may be an optimal and favorable laryngeal preservation strategy in resectable LAHPC.

## Notes

Supplement. **eFigure 1.** Treatment outcomes in different groups **eFigure 2.** The overall s survival and survival with functional larynx of different groups among patients without induction chemotherapy **eTable 1.** Clinical characteristics before and after PSM stratification by treatment strategies **eTable 2.** Key trials of laryngeal preservation strategies
