# Supplementary material for: Response-Adapted Treatment Following Radiotherapy in Patients With Resectable Locally Advanced Hypopharyngeal Carcinoma
Source: JAMA Netw Open. 2022 Feb 22;5(2):e220165. doi: 10.1001/jamanetworkopen.2022.0165 (PMC8864511; doi:10.1001/jamanetworkopen.2022.0165)
Supplement: Supplement. — eFigure 1. Treatment outcomes in different groups eFigure 2. The overall s survival and survival with functional larynx of different groups among patients without induction chemotherapy eTable 1. Clinical characteristics before and after PSM stratification by treatment strategies eTable 2. Key trials of laryngeal preservation strategies [file jamanetwopen-e220165-s001.pdf]

Supplemental Online Content

Luo X, Huang X, Liu S, et al. Response-adapted treatment following radiotherapy in patients with resectable locally advanced hypopharyngeal carcinoma. *JAMA Netw Open*. 2022;5(2):e220165. doi:10.1001/jamanetworkopen.2022.0165

eFigure 1. Treatment outcomes in different groups
eFigure 2. The overall survival and s survival with functional larynx of different groups among patients without induction chemotherapy
eTable 1. Clinical characteristics before and after PSM stratification by treatment strategies
eTable 2. Key trials of laryngeal preservation strategies

This supplemental material has been provided by the authors to give readers additional information about their work.

© 2022 Luo X et al. *JAMA Network Open.*

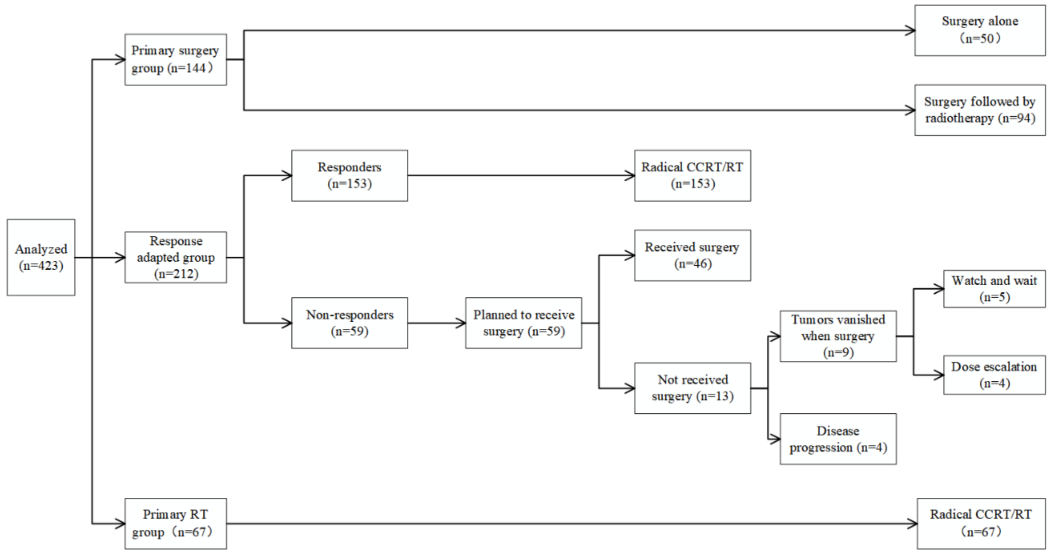

eFigure1. Treatment outcomes in different groups

Abbreviations: CCRT: chemoradiotherapy; Responders: patients who reached major PR at DT 50 Gy; Non-responders: the remaining patients.

© 2022 Luo X et al. *JAMA Network Open.*

eFigure2. The overall survival (A) and s survival with functional larynx (B) of different groups among patients without induction chemotherapy (n=347)

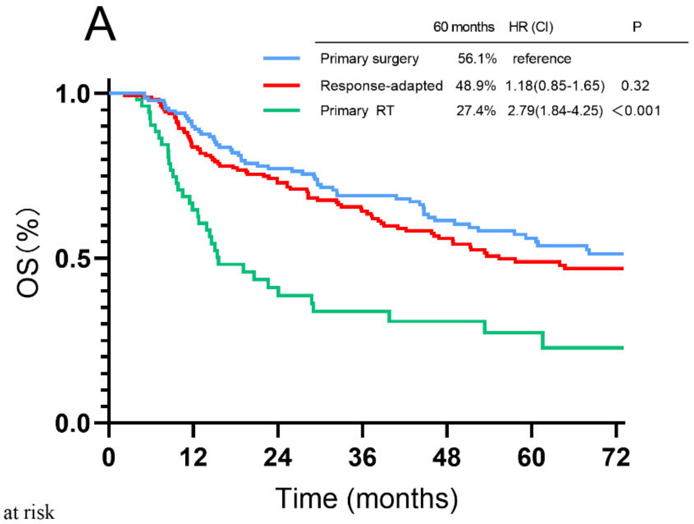

Patients at risk							
Primary surgery	135	116	96	81	63	49	41
Response-adapted	160	134	113	92	68	53	40
Primary RT	52	33	18	13	11	7	4

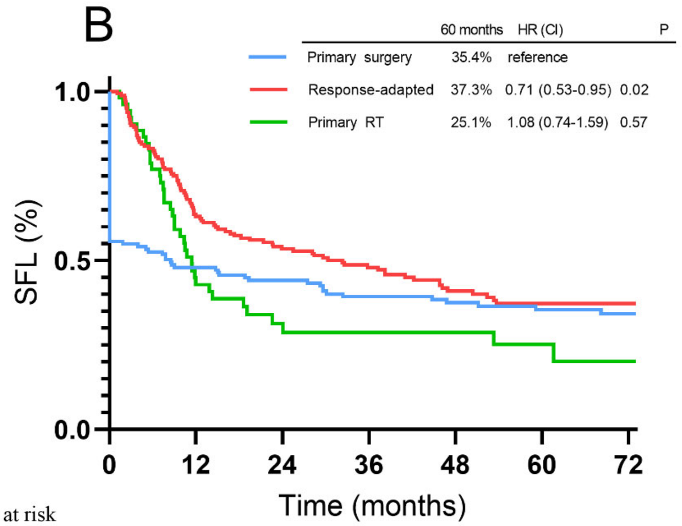

Patients at risk							
Primary surgery	135	63	57	49	41	32	27
Response-adapted	160	101	83	69	47	36	27
Primary RT	52	22	13	10	10	6	4

© 2022 Luo X et al. *JAMA Network Open.*

eTable1. Clinical characteristics before and after PSM stratification by treatment strategies

	**Before PSM（No.%）**			**After PSM（No.%）**		
	primary surgery	Response-adapted	P	primary surgery	Response-adapted	P
Total	144	212		121	121	
Sex			.78			.73
Male	140(97.2)	205(96.7)		117(96.7)	116(95.9)	
Female	4(2.8)	7(3.3)		4(3.3)	5(4.1)	
ECOG			.26			.84
0	22(15.2)	24(11.3)		14(11.6)	15(12.4)	
1	121(84.0)	188(88.7)		107(88.4)	106(87.6)	
2	1(0.8)	0(0)		0(0)	0(0)	
ENE (+)	13(9.0)	46(21.7)	.002	10(8.3)	10(8.3)	1.00
cT stage (AJCC 8th)			.48			.74
T1-2	52(36.1)	64(30.2)		50(41.3)	45(37.2)	
T3	39(27.1)	66(31.1)		34(28.1)	39(32.2)	
T4a	53(36.8)	82(38.7)		37(30.6)	37(30.6)	
cN stage (AJCC 8th)			.008			.99
N0	26(18.1)	25(11.8)		17(14.0)	15(12.4)	
N1	15(10.4)	20(9.4)		15(12.4)	15(12.4)	
N2	90(62.5)	120(56.6)		79(65.3)	81(66.9)	
N3	13(9.0)	47(22.2)		10(8.3)	10(8.3)	
TNM (AJCC 8th)			.007			.77
III	26(18.1)	28(13.2)		26(21.5)	22(18.2)	
IVA	104(72.2)	137(64.6)		84(69.4)	89(73.6)	
IVB	14(9.7)	47(22.2)		11(9.1)	10(8.2)	

Abbreviations: ENE: extranodal extension;

© 2022 Luo X et al. *JAMA Network Open.*

eTable2: Key trials of laryngeal preservation strategies

Trial	No. of patients	Enrollment patients	Stage	Treatment arms	Overall survival	Larynx preservation
EORTC 24,891	202	Hypopharynx (100%)	II-IV	S vs PF followed by RT	32.6% vs 38.0% (5yr)	22% (PF followed by RT group, SFL, 5yr)
EORTC 24,954	450	Hypopharynx (51%) and larynx (48%)	III-IV	Sequential vs alternating	48.5% vs 51.9% (5yr)	30.5% vs 36.2% (SFL, 5yr)
GORTEC 2000-01	213	Hypopharynx (54%) and larynx (46%)	III-IV	TPF followed by RT vs PF followed by RT	50.9% vs 41.9% (5yr)	74.0% vs 70.3% (LP rate, 5yr)
TREMPLIN	156	Hypopharynx (41%) and larynx (59%)	III-IV	primary RT vs BRT	75% vs 73%(3yr)	79% vs 72% (LDFS, 2yr)
DeLOS-II	180	Hypopharynx (50%) and larynx (50%)	II-IV	TPF/TP+R vs TPF/TP+R+E	68.2% vs 69.3% (2yr)	47.1% vs 46.6% (LFS, 2yr)
Beijing Tongren Hospital	260	Hypopharynx (100%)	III-IV	TPF followed by RT	32.6% (5yr)	24.8% (LDFS, 5yr)
Our study	432	Hypopharynx (100%)	III-IV	primary surgery vs Response-adapted vs primary RT	54.4% vs 52.7% vs 27.7% (5yr)	33.9% vs 40.6% vs 27.5% (SFL, 5yr)

Abbreviation: S:surgery; PF：cisplatin (P) and 5-fluorouracil (F); TPF：docetaxel (T), cisplatin (P) and 5-fluorouracil (F); RT: radiotherapy; primary RT：chemoradiotherapy; BRT bioradiotherapy；LP rate: larynx preservation rate; SFL: survival with functional larynx; LFS：survival with preserved larynx；LDFS: laryngoesophageal dysfunction-free survival.

© 2022 Luo X et al. *JAMA Network Open.*
